# Clinical and Forensic Investigation Protocols for Diagnosing Abusive Head Trauma: A Literature Review

**DOI:** 10.3390/diagnostics13193093

**Published:** 2023-09-29

**Authors:** Matteo Antonio Sacco, Saverio Gualtieri, Lucia Tarda, Pietrantonio Ricci, Isabella Aquila

**Affiliations:** Institute of Legal Medicine, Department of Medical and Surgical Sciences, “Magna Graecia” University, 88100 Catanzaro, Italy; matteoantoniosacco@gmail.com (M.A.S.); saveriogualtieri@icloud.com (S.G.); lucia.tarda@studenti.unicz.it (L.T.); ricci@unicz.it (P.R.)

**Keywords:** child abuse, abusive head trauma, forensic investigations

## Abstract

Abusive head trauma (AHT) represents a very serious global public health problem. Prevention of these episodes is essential to reduce the morbidity and mortality of this phenomenon. All healthcare professionals should be able to recognize the signs of abuse. However, diagnosis is very complex as the signs are often blurred and cannot be recognized with certainty without carrying out adequate instrumental investigations. It has been calculated that approximately one-third of AHT cases remain undetected and require more than one medical visit to be correctly interpreted and diagnosed. On the other hand, the literature has recently also emphasized the problems related to possible false diagnoses of abuse and the numerous family and personal repercussions that follow from this issue. For these reasons, correct and timely recognition is essential to avoid the risk of recurrence of AHT and to start proper forensic investigations, in order to identify the offender or exonerate a suspect. The present work explores the most recent evidence of recent years in the field of AHT diagnostics through a literature review. The purpose of this article is to provide forensic pathologists with clear tools for diagnosis based on the literature. To this end, the review suggests clinical and forensic protocols aimed at the timely diagnosis of AHT in order to prevent abuse from remaining undetected.

## 1. Introduction

### 1.1. Epidemiology of AHT

Child abuse is a serious emergency in the world. Abusive Head Trauma (AHT) has an incidence of approximately 20–40 children for every 100,000 births, therefore it is a complex phenomenon that represents an important cause of mortality and traumatic morbidity in childhood [1]. In Germany, between 100 and 200 children are hospitalized for AHT each year. Of these, up to 30% unfortunately do not survive due to the severity of the trauma suffered [2,3].

AHT is the leading cause of childhood traumatic brain injury (TBI) mortality. The big problem related to AHT is the often presence of vague physical signs and the choice to perform second-level instrumental investigations depends on the operator’s expertise and therefore on subjective parameters [4,5]. It has been calculated that approximately one-third of AHT victims have undiagnosed brain injuries and the diagnosis of abuse requires at least three visits. According to some authors, a large number of unexplained disabilities in children could be attributed to an incorrectly identified history of AHT. The lack of recognition of child abuse represents a very serious problem as it could lead to a repetition of the abuse. In a large number of cases, the lesions are clinically occult and the collection of the anamnesis is very difficult since these cases involve infants [6]. Crying is the typical trigger for shaking [7]. The most frequent answers given by perpetrators about the reasons for abuse are family problems, poor tolerance, and dyscontrol of impulses [8].

### 1.2. False Positives Issue

Over the past decade, there has been much controversy regarding the diagnosis of child abuse. In particular, several authors have highlighted the possibility of false accusations of AHT and the inadequacy of diagnostic protocols for abuse. To date, in fact, the debate is still open, especially regarding the risk of false positives and incorrect diagnoses. In particular, the literature discusses which signs should be useful for diagnosing abuse, and how these limits can be overcome. Several authors have highlighted the need to reduce the risk of bias, favoring on the one hand the importance of confessions, carried out with methodological criteria, and on the other hand, discussing the actual diagnostic sensitivity and specificity of other signs, such as retinal hemorrhage, on the topic of AHT [9,10]. Therefore, it is essential to reduce the incidence of false positives because this can have repercussions not only for children but also for parents who, if wrongly accused, can lose custody of their child. The literature has analyzed the phenomenon of post-traumatic stress syndrome in unjustly accused parents and the development of a lack of trust in the healthcare system and judicial authorities. In addition to the classic diagnostic triad (retinal hemorrhage, subdural lesions, and neuronal damage), this study describes further elements that can help both clinical and forensic pathologists lean toward the correct diagnosis. Therefore, the aim of this review is to analyze all the data available in the literature by proposing a protocol applicable to patients and in post-mortem forensic investigations and emphasizing correct diagnostics in order to avoid errors that can have repercussions on entire families and personal and social units.

### 1.3. Terminology

Abuse with physical violence has been described since the 19th century when the pathologist Tardieu recognized a typical pattern characterized by meninges hemorrhages, with brain damage. In the 1970s, the existence of a real syndrome was proposed for the first time, defined as Whishplash Shaken Infant Syndrome, due to shaking movements of the head, with violent acceleration, traction, rotation, and deceleration to determine a characteristic typical clinical and topographic pattern [11]. In the 1980s, this pattern was called Shaken Baby Syndrome (SBS). Other terms used include “battered child syndrome” and “parent-infant traumatic stress syndrome” [6]. However, in 2009, the American Academy of Pediatrics (AAP) introduced the term Abusive Head Trauma (AHT) to define a complex syndrome showing head trauma and/or intracerebral lesions in a child under the age of 5 due to a violent shaking movement or using blunt objects against the head [12]. The term AHT therefore includes all craniospinal injuries involving the head following SBS or blunt force attack to the head or a combination of both. Diagnostic accuracy measures the percentage of correct diagnoses, i.e., how many patients are correctly classified as abused. In this paper, we also considered the term sensitivity as the ability to identify abused subjects and specificity as the identification of healthy subjects, i.e., not actually abused. It is important to consider that the described diagnostic findings are medical observations performed by the various authors, so the conclusion that they were caused by intentional violence is an inference of the unobserved circumstances that led to the injuries.

### 1.4. Pathophysiology of AHT

The exact pathophysiology of AHT-associated injuries is still under investigation. A fundamental role seems to be assumed by injuries affecting the meningeal vessels and the arachnoid with the production of subdural lesions consisting of blood, blood derivatives, and collections of cerebrospinal fluid [13,14,15]. The main mechanism is therefore the rupture of the bridging vein vessels through acceleration, deceleration, and angular rotation movements due to head shaking. In addition to subdural hematomas, it is possible to have axonal lesions due to the tearing of the white and gray matter, but also retinal lesions. These injuries are more frequent in children due to the larger size of the head compared to the body, the high water content, poor control of head movements, and the myelination process still not being complete [16,17,18]. Among the various hypotheses to explain the phenomenon of retinal hemorrhages, the most accredited would concern tearing of the vessels at the level of the adhesion structures between the vitreous and retina, papilla-retina, and macula-vitreous. In particular, the exact mechanisms and the force necessary to produce brain lesions are still being studied today. A recent experimental study of biomechanics analyzed the force and mechanisms during shaking in a laboratory model with a device compatible with a child weighing 3.5 kg with accelerometers on the head, asking volunteers to shake the object vigorously [19]. Investigations showed that the violent shaking movement of the head can induce high-energy tangential accelerations with high centripetal force. These mechanisms could increase the pressure of the subdural cranial district producing an encephalic compression with violent tearing of the bridging veins. In newborns, the brain is in fact contained within a box with incomplete ossification. The weight of the brain also represents 10–15% of the total weight of the infant, while in adults, it is equal to 2–3%. The water content is very high, and the nervous structures are immature and fragile. Furthermore, the weakness of the neck muscle groups determines greater exposure to exogenous acceleration and deceleration movements [20].

## 2. Materials and Methods

A literature review was performed using the Pubmed NCBI and Scopus Embase search engines on the diagnostics of AHT. The following keywords were used: pediatric abusive head trauma OR shaken baby syndrome AND forensic. The research included English papers concerning clinical, instrumental, and forensic investigations used in the context of AHT.

## 3. Results and Discussion

### 3.1. Ophtalmological Investigations

The search for retinal hemorrhages is essential for the diagnosis of abuse. The literature revealed that a high blood point count is highly suggestive of AHT. In one study, it was evaluated that the collection of 25 dot-blood counts is associated with a greater than 90% probability of AHT [21]. Moskwa et al. evaluated a sample of 133 AHT cases in a retrospective descriptive analysis [21]. The authors investigated the various types of eye lesions, their localization, and their correlation with age and gender. The authors reported the presence of ophthalmological lesions in 70% of the series of which the vast majority (approximately 95%) were retinal hemorrhages, while other possible ocular lesions were papilledema and vitreous hemorrhage. In approximately 80% of cases, the lesions were bilateral. Fundus evaluation in cases of suspected AHT abuse is therefore mandatory. Observation of the retina requires careful evaluation with dilated pupils by an ophthalmologist. The severity of retinal hemorrhages is also strongly correlated with neurological damage, typically with a bilateral distribution.

Backdating eye injuries still remains complicated. The resorption time of intraretinal hemorrhagic lesions can reach two weeks, while the presence at the preretinal level can persist for several months. The differential diagnosis of these injuries requires the exclusion of accidental traumatic injuries or injuries related to childbirth [21,22,23].

The mechanisms behind retinal hemorrhage are not fully understood, and two main theories have been postulated, of which the first concerns vitreoretinal traction related to shaking movements. The second mechanism concerns the increase in intracranial pressure from intracranial hemorrhage with increased ocular venous return and the production of hemorrhages. This mechanism could also occur in cases of increased intrathoracic pressure, as in the case of chest compressions during resuscitation maneuvers, but this pathogenesis appears more unlikely. A systematic review of the literature examining over 60 studies in this area reported that the likelihood of child abuse showing head trauma and retinal hemorrhage is 91%, even if the majority of the data were collected by case studies [24]. Furthermore, there would seem to be a correlation between the severity of retinal lesions and the severity of brain damage. The location of retinal lesions can vary, as well as their size and severity. Localization can be peripapillary, on the posterior pole (most frequent site), and on the more peripheral ora serrata. Most of the studies have described the involvement of the more superficial retinal layers, i.e., the subinternal limiting membrane and the ganglion cells. On the other hand, the vitreous localizations are rarer, and more often found at autopsy and in post-mortem investigations [25].

### 3.2. Radiological Investigations

In Germany, Hahnemann et al. performed a multicenter study analyzing neuroimaging material from 56 children, revealing the multifocal presence of subdural collections in over 96% of cases. The most frequent radiological entity was subdural hematohygroma (66%) proximal to subdural collections of cases with signs of recent head injury [1].

When abuse is suspected, a radiological evaluation is therefore indispensable both for the diagnosis and evaluating acute or chronic patterns of injury, along with evaluation of the subdural neomembranes (chronic subdural hematoma) of which analysis can suggest the repetitiveness of the violence. In a systematic review, Kadom et al. described the presence of subdural lesions visible on neuroimaging in cases of suspected abuse with an odds ratio of 8.2% [26]. Analysis with neuroradiology generally allows one to visualize subdural hematoma in the context of cerebral compression lesions of the sulcus and dislocation of corticodural venous vessels. [27]. The contextual finding of intraparenchymal lesions and fractures is also possible.

Several works have also demonstrated the involvement of the spinal cord when examined with a CT scan or MRI investigation. Koumellis et al. analyzed the results of a series of 18 AHTs in children aged 3 months admitted to a neuroscience center, noting that when the neuroradiological investigation was performed on the entire spinal column, subdural spinal hematoma was observed in approximately half of the cases (44%) [28].

CT has a high sensitivity for identifying bone tissues and is essential for identifying fracture lines, areas of cerebral edema, and signs of ischemic or hemorrhagic lesions. An MRI would instead represent the ideal neuroradiological investigation to analyze AHT injuries as it is characterized by greater sensitivity. According to recent guidelines, CT should be carried out in cases of suspected AHT with 3D reconstructions, followed by an MRI investigation of the brain. MRI should be performed repeatedly since parenchymal and extra-axial lesions may not be visible in the first instance [29]. The ideal sequences for injury visualization are GRE T2-w and SWI, which can identify blood collections and venous vessel lesions. The typical sign of subdural lesions is the Tapdole Sign, where the body is represented by a clot derived from the injured veins, extended by the coagulated blood.

Backdating bleeding lesions can be extremely complex as there are no defined rules in MRI evolution. Bradford et al. identified different patterns of SDH in a group of 43 infants, reporting that homogeneous SDH corresponds to early or subacute MR findings while heterogeneous SDH is represented by a set of different intensities [30]. Subdural hygroma represents the final stage and can be considered the terminal stage of subdural hemorrhage. Subarachnoid hemorrhage is a nonspecific finding, likely caused by tearing of the vessels of the pia mater and the arachnoid. The recognition of subarachnoid hemorrhage and subdural hygroma is often complicated, and both lesions may even coexist [29].

### 3.3. Brain Injuries

A key diagnostic element is the presence of subdural collections. In particular, it is possible to find subdural hemorrhage, subdural hygroma, subdural hematohygroma, and chronic subdural hematoma. Subdural hematoma is more common in infants than in children of other ages. In a recently published work, a group of 37 children who presented with subdural hematoma were compared, 20 of whom were cases of confirmed AHT while 17 were cases attributable to another cause [31]. The authors found an association between AHT and symptoms like epilepsy, altered state of consciousness, retinal hemorrhages, fractures, intrahemispheric blood, blood in the posterior cranial fossa, and bilateral brain lesions compared to the control group. Another type of injury, less investigated than the others, is represented by external hydrocephalus. A significant proportion of children with chronic subdural hematoma also have signs of external hydrocephalus.

The spinal cord is also a frequent site of injury. The literature shows that an examination of the spinal cord allows the identification of bleeding lesions in over 40% of cases. Several studies have demonstrated the presence of extra-axial bleeding in cases of AHT. The results were confirmed in the case studies reviewed by Dashti et al. who analyzed 32 AHT children under two years old and in the study by Vinchon et al. who reported signs of subdural hemorrhage in 82% of the case studies examined, in comparison with the control groups [32,33].

**Figure 1:**
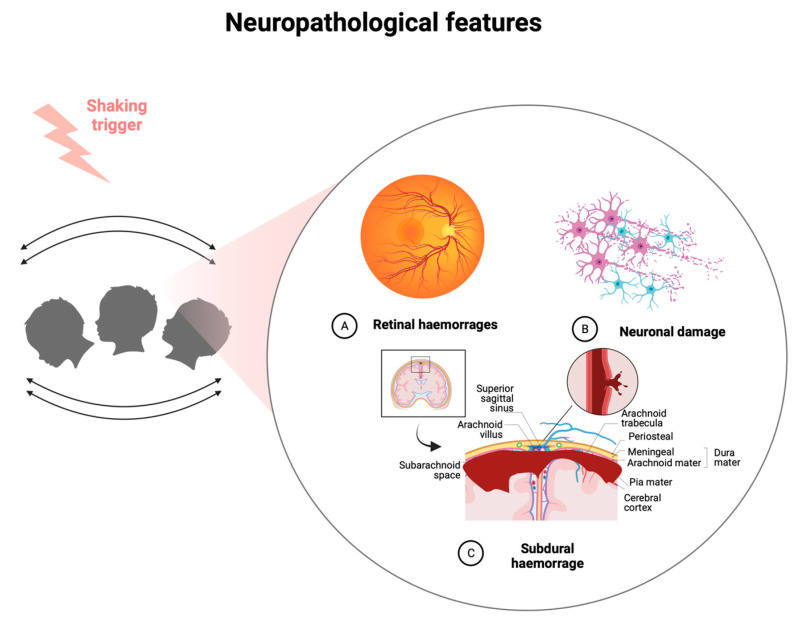
Neuropathological features of AHT (created with BioRender.com, (accessed on 26 September 2023)).

Cerebral edema with hypoxic ischemic lesions is frequently associated with abuse. In particular, several authors have shown a possible association between cerebral edema and AHT [34]. Among other injuries, it is possible to find intraparenchymal lesions, cerebral contusions and lacerations, and fractures of the skull. The bridging veins are located at the vertex level and on the arachnoid space perpendicular to the superior sagittal sinus, with particular susceptibility to anteroposterior oscillation movements (Figure 1).

### 3.4. Laboratory Investigations

In the laboratory setting, there are no defined laboratory screening tests that are helpful in making the AHT diagnosis. In recent years, there has been growing interest in protein analysis in forensic pathology. Wiskott et al. examined the proteome in a series of 52 samples divided into two groups (ante-mortem and post-mortem), each of which was divided into cases and controls [35]. The investigations were carried out through Liquid chromatography-electrospray ionization tandem mass spectrometry (LC-ESI-MS/MS). Serum investigations in ante-mortem samples demonstrated the upregulation of several brain trauma-specific proteins compared to controls, such as glutathione S-transferase LANCL1 (LANCL1), Apolipoprotein J, and glycolytic enzyme Alpha-mannosidase 2 (MAN2A1). In particular, three proteins were upregulated related to brain development, namely Autotaxin (ATX), Neural cell adhesion molecule L1-like protein (CHL1), and BASP 1. ATX is an enzymatic protein involved in the synthesis of lysophosphatidic acid essential in neuronal development, produced in the cerebrospinal fluid. CHL1 is a cell adhesion molecule expressed in neurons and glial cells, especially in the cerebral cortex implicated in the development of the nervous system, neuron growth of the cerebellum and hippocampus, and post-traumatic neuronal regeneration. Finally, BASP1 showed elevated levels in both ante-mortem and post-mortem samples. This protein shows high concentrations during brain development, decreasing its concentrations with age in an inversely proportional manner. The investigations demonstrated the possibility of identifying proteomic screening methods in cases of suspected AHT through the search for potential biomarkers of brain damage. Therefore, the search for potential biomarkers of brain damage is highly desirable in order to improve the early diagnosis of these events, even when not clinically detectable in the first instance [35] (Table 1).

### 3.5. Medico Legal Investigations on Child and Post-Mortem Analysis

In the face of numerous studies focused on brain and retinal lesions, few studies have been conducted on injuries of other types, such as skeletal or skin lesions. Feld et al. analyzed a series of 72 cases of children undergoing a coroner’s examination in Germany [36]. Fractures were found in approximately 30% of cases, especially in the clavicle and skull. Skin lesions consisting of hematomas or abrasions were found in approximately 50% of cases, concentrated on the face, head, and trunk. The data show how AHT is often accompanied by a form of physical abuse, which is also characterized by extracerebral lesions. Therefore, a detailed forensic examination is necessary, accompanied by a skeletal survey if abuse is suspected.

At autopsy, intraocular hemorrhages are very frequent in cases of suspected abuse, with a sensitivity and specificity of 75% and 94%, respectively [37]. The assessment of AHT should not be based solely on a single sign but on a series of evidence [37]. In several autopsy cases, post-mortem enucleation is described for evaluation of the cause of death. In recent works, the possibility of ophthalmoscopy on cadavers has also been evaluated, making use of photographic image technologies. However, post-mortem evaluations of the eye with the OCT exam have limitations related to the corneal opacity, due to post-mortem processes for which more studies are needed in this regard. A recent systematic literature review on AHT examined the role of post-mortem CT, signaling the role that this investigation plays in post-mortem diagnostics. It is useful for the identification of skull fractures and internal bleeding, which represent the most frequent findings in cases of abuse [38].

Histopathologic examination may demonstrate hemorrhagic cleft, subhyloid hemorrhage, and subretinal hemorrhage. It is also possible to evaluate intraretinal hemorrhages (cherry pattern), intrascleral hemorrhage, and subdural hemorrhage of the optic nerve. One of the main signs is represented by the identification of the cherry hemorrhage in the internal-limiting membrane. A literature review demonstrated that immunohistochemistry could demonstrate axonal damage using the beta-amyloid precursor protein staining (βAPP) marker [39]. An adequate interpretation of the immunohistochemical results is necessary as the positivity to this marker may also be present in other cases such as in hypoxic/ischemic, metabolic, and traumatic insults. Glycophorin-A, an erythrocyte membrane protein, has also been suggested as a useful marker for detecting hemorrhagic lesions. A recent experimental study examined a series of 113 cases under 2 years of age, of which 8 cases resulted in death [20]. Post-mortem investigations demonstrated subdural and subarachnoid hematoma and intracranial hemorrhage in 50% of cases. Microscopic histopathological examination instead showed signs of focal subdural bleeding in 50% of cases localized at the spinal cord level. In all cases, retinal hemorrhage was evident, which occurred with bilateral localization in 75% of cases.

### 3.6. Investigation Protocols for Diagnostics

#### 3.6.1. Clinical Investigation Protocols

**Figure 2:**
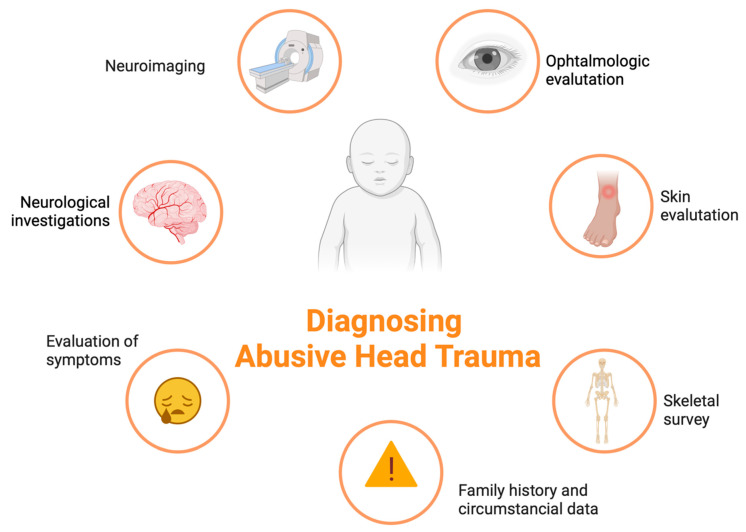
Diagnostics protocol for AHT (created with BioRender.com, (accessed on 26 September 2023)).

The classic pathological triad should be intracranial subdural hematoma, encephalopathy with ischemic hypoxic damage, and retinal hemorrhages (Figure 1). However, several authors dispute the diagnostic value of this triad. In the clinic, AHT should always be suspected when a newborn/child shows evidence of a head injury or signs and symptoms of head injury (headache, epilepsy, drowsiness, etc.). The evaluation should always consider investigation protocols according to a flowchart approach through careful examination with the intervention of a forensic expert and a pediatrician, ophthalmological evaluation with fundus examination, the performance of level I (brain X-ray with the evaluation of the fontanelles) and II (CT/MRI) radiological investigations, the exclusion of alternative diagnoses, and the evaluation of laboratory tests (Figure 2). The history should always include a thorough collection of data provided by the parents on the event, with the evaluation of any circumstantial data if available (history of past abuse, drug abuse, etc.) and, where possible, a history of the child in the presence of a pediatrician. Suspicion should be increased when simultaneous fractures are found, especially if they are multiple and in different anatomical sites [40].

#### 3.6.2. Post-Mortem Investigation Protocols

Post-mortem investigations must include timely analyses. The investigations should start with the evaluation of circumstantial data from the parents or those who assisted the child, with the collection of testimonies from neighbors or acquaintances. The investigation should always include the analysis of the scene with a direct inspection aimed at collecting evidence of abuse. In all cases, CT should be performed before the autopsy, with 3D reconstruction sequences of the head. This investigation is essential in order to evaluate any lesions on which to focus even before the autopsy. Furthermore, this assessment is essential to radiologically crystallize any injury that should be altered during autopsy. Particular attention should be paid to signs of brain injury.

Autopsy must be considered mandatory in all doubtful or suspicious cases. The examination should include an accurate external analysis with photos. All external injuries (grazes, bruises, burns, lacerations, etc.) should be photographed and measured with a color reference [40]. Ecchymoses should be incised to evaluate their vitality and taken to allow a microscopic visualization. The forensic pathologist must therefore carry out an accurate examination of the brain with the removal of the entire organ. The cranial fossae must be visualized carefully as it is possible that they are more difficult to visualize on CT. The pathologist should also perform an OCT in order to visualize the retina fundus and subsequently proceed to the enucleation for the study in its entirety. Spinal cord examination at autopsy is essential. The histopathological examination must always be performed in order to exclude any alternative disease and to obtain a microscopic analysis of the injuries. The contribution of immunohistochemistry with the search for markers such as glycophorin can prove to be of support in the diagnosis.

#### 3.6.3. Forensic Investigation of the Perpetrator

Defining responsibility for abuse can be extremely complicated. In 50–60% of cases, the father is responsible, in 9% of cases, the mother’s partner is responsible, and in approximately 30% of cases, it is the mother herself. A recent study showed that 50% of cases in Germany involving suspected abuse cases were closed without trial due to the lack of evidence against a specific person [36]. In another study, Clauß et al. found that in 33% of the cases examined, the proceedings were terminated by the Public Prosecutor due to a lack of suspects [42]. The main problems include the lack of sufficient evidence from the prosecution, the lack of testimony, and the impossibility of obtaining information from the child. It is clear that timely recognition of AHT is essential to start a forensic investigation and find the perpetrator.

#### 3.6.4. Differential Diagnosis with Pathologies Not Related to Abuse

An accurate differential diagnosis is always necessary in cases of suspected child abuse. This diagnosis must take into account numerous other possible diseases such as congenital vascular malformations, infections of the nervous system, coagulation disorders, accidental head trauma, and galactosemia. Unuma et al. described a complex case of death of a two-month-old infant presenting with subdural hematoma, retinal hemorrhage, and encephalopathy, without any fractures [41]. Histopathological examination, however, showed B-cell acute lymphoblastic leukemia for which after a detailed differential diagnostic investigation, AHT was excluded.

## 4. Conclusions

### Conclusions and Future Research Perspectives

The challenges related to this issue concern the timing of recognition of AHT at the first medical contact. In suspected cases, it is desirable to apply clear and defined algorithms that support the decisions of the clinician to first interface with the case. The diagnosis requires the intervention of a multidisciplinary team, which must necessarily include the presence of a forensic expert, a pediatrician, an ophthalmologist, and a radiologist in a global assessment that requires multiple skills. The challenges related to the clinical setting concern the improvement of the sensitivity and specificity of the diagnostic algorithms and the search for potential early biomarkers correlated to AHT.

The evaluation of a case of suspected AHT requires an overall analysis of the cerebral pathological and neuroimaging evidence, accompanied by the collection of family history with an evaluation of risk factors, a careful external examination with a search for skin lesions, the evaluation of fractures, and the exclusion of comorbidities or alternative diagnoses. Forensic investigations must be promptly started in suspected cases, with a warning sent to the Judicial Authority, the collection of testimonies and investigations by electronic support, and surveillance strategies for suspects. The autopsy accompanied by the CT scans must be considered mandatory in all suspected cases or those with incongruous traumatic dynamics. This study emphasizes how and when diagnostic investigations such as ophthalmological evaluation, neuroimaging, a skeletal investigation, a thorough external skin examination, the evaluation of symptoms, and an in-depth neuronal investigation of the child are consistent with the diagnosis of abuse, as well as how careful circumstantial data, high levels of diagnostic sensitivity, and specificity can be achieved.

## Figures and Tables

**Table 1:** Summary of literature review results.

Authors	Type of Study	Number of Suspected or Confirmed AHT Cases Analysed	Investigations Performed	Results
Hahnemann et al. [1]	Retrospective study	56	Neuroimaging analysis	96.4% showed a multifocal presence of SDCs
Moskwa et al. [21]	Retrospective study	133	Ophthalmological analysis	Ophthalmologic lesions were in 70.3%
Yamazaki et al. [22]	Experimental study		Experimental model with a doll	Time integral of stress in shaking is 107 Pa.s, larger than that of a fall
Yoshida et al. [23]	Experimental study		Experimental model with a finite element	Time integral of stress in shaking is 101 Pa.s, larger than that of a single impact
Maguire et al. [24]	Systematic review	998	Ophthalmological analysis	Retinal hemorrhages were found in 78% of AHT.
Kadom et al. [26]	Retrospective study	64	Neuroimaging analysis	36% of children showed cervical spine injuries. A statistically significant relationship was found between bilateral hypoxic–ischemic brain injury patterns and AHT
Vinchon et al. [27]	Prospective study	1138	Review of registry	Physicians should exclude non-traumatic bleeding and second, accidental trauma. A systematic and multidisciplinary approach is mandatory.
Koumellis et al. [28]	Retrospective study	18	Neuroimaging analysis	44% of cases showed occult subdural collections
Cartocci et al. [29]	Review	-	Neuroimaging analysis	Radioimaging is mandatory in suspect of AHT. Subdural hemorrhages with bridging vein rupture and thrombosis are more commonly associated
Bradford et al. [30]	Retrospective study	210	Neuroimaging analysis	In patients with subdural hematomas, the first hypodense component due to SDH was between 0.3 and 16 days after injury, and the last hyperdense component disappeared between 2 and 40 days after injury.
Snelling et al. [31]	Retrospective study	20	Clinical and radiological analysis	SDH was associated with maternal drug use, delayed presentation in ED, seizures, altered level of consciousness; fractures, skin injuries or retinal hemorrhages; radiological findings of bilateral and inter-hemispheric blood
Dashti et al. [32]	Retrospective study	38	Clinical and radiological analysis	SDH was in 69% of cases, retinal hemorrhages were in 53% of cases. Low socioeconomic status was associated with SDH.
Vinchon et al. [33]	Retrospective study	45	Clinical and radiological analysis	SDH, severe RH, and absence of signs of impact were associated with AHT
Keenan et al. [34]	Retrospective study	80	Clinical and radiological analysis	RH, fractures, and SDH were more associated with AHT
Wiskott et al. [35]	Experimental study	7	Proteomic analysis	165 circulating serum proteins display differences in AHT cases versus controls
Feld et al. [36]	Retrospective study	72	Clinical and radiological analysis	Fractures were found in 32% of cases; skin injuries were found in 53% of cases
Bhardwaj et al. [37]	Systematic review	20 studies	Ophthalmological analysis	Bilateral, extensive, and intraocular hemorrhages are highly specific for AHT
Colombari et al. [38]	Review	-	Radiological and neuropathological spinal cord examination	Spinal blood collection is the most indicative finding in AHT.
Maiese et al. [39]	Systematic review	49 studies	Clinical and forensic investigations	circumstantial data, post-mortem radiological examinations, autopsy, and histological examination of the eye and brain play an important role
Oruç et al. [20]	Retrospective study	8	Clinical and radiological analysis	The main associated findings were: male sex, trauma perpetrated by the father, bilateral retinal hemorrhage
Messing-Jünger et al. [40]	Case report	1	Clinical and radiological analysis	massive bilateral retinal bleedings, fracture of the femur, left SDH
Unuma et al. [41]	Case report	1	Autopsy and post-mortem histological examination	Subdural hematoma, retinal hemorrhage, and encephalopathy without fractures were present but post-mortem examination revealed acute lymphoblastic leukemia

## Data Availability

Not applicable.

## References

[1] Hahnemann M.L., Kronsbein K., Karger B., Feld K., Banaschak S., Helmus J., Mentzel H.-J., Pfeiffer H., Wittschieber D. (2023). Characterization of subdural collections in initial neuroimaging of abusive head trauma: Implications for forensic age diagnostics and clinical decision-making. Eur. J. Radiol..

[2] Beam A.S., Stephens C.P., Taylor C., Bentley J., Gonzalez A.C., Marwaha M., Riley D., Wade C. (2023). Imaging and Demographic Risk Factors in the Diagnosis of Pediatric Nonaccidental Trauma. Radiol. Technol..

[3] Emrick B.B., Smith E., Thompson L., Mullett C., Pino E., Snyder K., Kroll M.-A., Ayoubi S., Phillips J., Istfan S. (2019). Epidemiology of abusive head trauma in West Virginia children <24 months: 2000–2010. Child Abuse Negl..

[4] Macorano E., Gentile M., Stellacci G., Manzionna M., Mele F., Calvano M., Leonardelli M., Duma S., De Gabriele G., Cristalli A. (2023). ‘Compressed Baby Head’: A New ‘Abusive Head Trauma’ Entity?. Children.

[5] Knappstein J., Reed P.W., Kelly P. (2023). ICD-10 codes for surveillance of non-fatal abusive head trauma in Aotearoa New Zealand: A retrospective cohort study. BMJ Open.

[6] Pfeifer C.M., Henry M.K., Caré M.M., Christian C.W., Servaes S., Milla S.S., Strouse P.J. (2021). Debunking Fringe Beliefs in Child Abuse Imaging: AJRExpert Panel Narrative Review. AJR Am. J. Roentgenol..

[7] Wiley M., Schultheis A., Francis B., Tiyyagura G., Leventhal J.M., Rutherford H.J., Mayes L.C., Bechtel K. (2020). Parents’ Perceptions of Infant Crying: A Possible Path to Preventing Abusive Head Trauma. Acad. Pediatr..

[8] Talvik I., Alexander R.C., Talvik T. (2008). Shaken baby syndrome and a baby’s cry. Acta Paediatr..

[9] Högberg U., Eriksson G., Högberg G., Wahlberg Å. (2020). Parents’ experiences of seeking health care and encountering allegations of shaken baby syndrome: A qualitative study. PLoS ONE.

[10] Lynøe N., Eriksson A. (2022). Agreements and disagreements regarding "shaken baby syndrome". Childs Nerv. Syst..

[11] Di Fazio N., Delogu G., Morena D., Cipolloni L., Scopetti M., Mazzilli S., Frati P., Fineschi V. (2023). New Insights into the Diagnosis and Age Determination of Retinal Hemorrhages from Abusive Head Trauma: A Systematic Review. Diagnostics.

[12] Christian C.W., Block R., Committee on Child Abuse and Neglect, American Academy of Pediatrics (2009). Abusive head trauma in infants and children. Pediatrics.

[13] Raghupathi R., Prasad R., Fox D., Huh J.W. (2023). Repeated mild closed head injury in neonatal rats results in sustained cognitive deficits associated with chronic microglial activation and neurodegeneration. J. Neuropathol. Exp. Neurol..

[14] Kato M., Nonaka M., Akutsu N., Narisawa A., Harada A., Park Y.-S. (2023). Correlations of intracranial pathology and cause of head injury with retinal hemorrhage in infants and toddlers: A multicenter, retrospective study by the J-HITs (Japanese Head injury of Infants and Toddlers study) group. PLoS ONE.

[15] Harris C.K., Stagner A.M. (2023). The Eyes Have It: How Critical are Ophthalmic Findings to the Diagnosis of Pediatric Abusive Head Trauma?. Semin. Ophthalmol..

[16] Miller D.C., Stacy C.C., Duff D.J., Guo S., Morse P. (2022). Neuropathology and Ophthalmological Pathology of Fatal Central Nervous System Injuries in Young Children: Forensic Neuropathology of Deaths of Children Under Age 2, 2008–2016, in Central Missouri. J. Neuropathol. Exp. Neurol..

[17] Finnie J.W., Blumbergs P.C. (2022). Animal models of pediatric abusive head trauma. Childs Nerv. Syst..

[18] Mavroudis I., Kazis D., Chowdhury R., Petridis F., Costa V., Balmus I.-M., Ciobica A., Luca A.-C., Radu I., Dobrin R.P. (2022). Post-Concussion Syndrome and Chronic Traumatic Encephalopathy: Narrative Review on the Neuropathology, Neuroimaging and Fluid Biomarkers. Diagnostics.

[19] Stray-Pedersen A., Strisland F., Rognum T.O., Schiks L.A.H., Loeve A.J. (2021). Violent Infant Surrogate Shaking: Continuous High-Magnitude Centripetal Force and Abrupt Shift in Tangential Acceleration May Explain High Risk of Subdural Hemorrhage. Neurotrauma Rep..

[20] Oruç M., Dündar A.S., Okumuş H., Görmez M., Şamdancı E.T., Celbiş O. (2021). Shaken baby syndrome resulting in death: A case series. Turk. J. Pediatr..

[21] Moskwa R., Todeschi J., Wiedemann-Fode A., Stella I., Joud A., Klein O. (2022). Ophthalmological lesions in shaken baby syndrome: A retrospective analysis of 133 consecutive cases (1992–2018). Neurochirurgie.

[22] Yamazaki J., Yoshida M., Mizunuma H. (2014). Experimental analyses of the retinal and subretinal haemorrhages accompanied by shaken baby syndrome/abusive head trauma using a dummy doll. Injury.

[23] Yoshida M., Yamazaki J., Mizunuma H. (2014). A finite element analysis of the retinal hemorrhages accompanied by shaken baby syndrome/abusive head trauma. J. Biomech..

[24] Maguire S.A., Watts P.O., Shaw A.D., Holden S., Taylor R.H., Watkins W.J., Mann M.K., Tempest V., Kemp A.M. (2013). Retinal haemorrhages and related findings in abusive and non-abusive head trauma: A systematic review. Eye.

[25] Ang J.L., Collis S., Dhillon B., Cackett P. (2021). The Eye in Forensic Medicine: A Narrative Review. Asia Pac. J. Ophthalmol..

[26] Kadom N., Khademian Z., Vezina G., Shalaby-Rana E., Rice A., Hinds T. (2014). Usefulness of MRI detection of cervical spine and brain injuries in the evaluation of abusive head trauma. Pediatr. Radiol..

[27] Vinchon M. (2017). Shaken baby syndrome: What certainty do we have?. Child’s Nerv. Syst..

[28] Koumellis P., McConachie N.S., Jaspan T. (2009). Spinal subdural haematomas in children with non-accidental head injury. Arch. Dis. Child..

[29] Cartocci G., Fineschi V., Padovano M., Scopetti M., Rossi-Espagnet M.C., Giannì C. (2021). Shaken Baby Syndrome: Magnetic Resonance Imaging Features in Abusive Head Trauma. Brain Sci..

[30] Bradford R., Choudhary A.K., Dias M.S. (2013). Serial neuroimaging in infants with abusive head trauma: Timing abusive injuries. J. Neurosurgery: Pediatr..

[31] Snelling P.J., Thanasingam A.A., Jones P., Connors J. (2022). Comparison of abusive head trauma *versus* non-inflicted subdural haematoma in infants: A retrospective cohort study. Emerg. Med. Australas..

[32] Dashti S.R., Decker D.D., Razzaq A., Cohen A.R. (1999). Current Patterns of Inflicted Head Injury in Children. Pediatr. Neurosurg..

[33] Vinchon M., de Foort-Dhellemmes S., Desurmont M., Delestret I. (2010). Confessed abuse versus witnessed accidents in infants: Comparison of clinical, radiological, and ophthalmological data in corroborated cases. Childs Nerv. Syst..

[34] Keenan H.T., Runyan D.K., Marshall S.W., Nocera M.A., Merten D.F. (2004). A Population-Based Comparison of Clinical and Outcome Characteristics of Young Children with Serious Inflicted and Noninflicted Traumatic Brain Injury. Pediatrics.

[35] Wiskott K., Gilardi F., Hainard A., Sanchez J., Thomas A., Sajic T., Fracasso T. (2023). Blood proteome of acute intracranial hemorrhage in infant victims of abusive head trauma. Proteomics.

[36] Feld K., Ricken T., Feld D., Helmus J., Hahnemann M., Schenkl S., Muggenthaler H., Pfeiffer H., Banaschak S., Karger B. (2022). Fractures and skin lesions in pediatric abusive head trauma: A forensic multi-center study. Int. J. Legal Med..

[37] Bhardwaj G., Chowdhury V., Jacobs M.B., Moran K.T., Martin F.J., Coroneo M.T. (2010). A Systematic Review of the Diagnostic Accuracy of Ocular Signs in Pediatric Abusive Head Trauma. Ophthalmology.

[38] Colombari M., Troakes C., Turrina S., Tagliaro F., De Leo D., Al-Sarraj S. (2021). Spinal cord injury as an indicator of abuse in forensic assessment of abusive head trauma (AHT). Int. J. Leg. Med..

[39] Maiese A., Iannaccone F., Scatena A., Del Fante Z., Oliva A., Frati P., Fineschi V. (2021). Pediatric Abusive Head Trauma: A Systematic Review. Diagnostics.

[40] Messing-Jünger M., Alhourani J. (2022). A suspected case of shaken baby syndrome-clinical management in Germany: A case-based overview. Childs Nerv. Syst..

[41] Unuma K., Makino Y., Yamamoto K., Hattori S., Arai N., Sakai K., Kitagawa M., Uemura K., Kanegane H. (2021). Fatal intracranial hemorrhage due to infantile acute lymphoblastic leukemia mimicking abusive head trauma. J. Forensic Sci..

[42] Clauß D., Richter C., Klohs G., Heide S. (2013). Strafprozessuale Folgen von Kindesmisshandlung [Legal consequences in cases of child abuse]. Klin. Padiatr..

